# Modeling spatial access to cervical cancer screening services in Ondo State, Nigeria

**DOI:** 10.1186/s12942-020-00222-4

**Published:** 2020-07-21

**Authors:** Kathleen Stewart, Moying Li, Zhiyue Xia, Stephen Ayodele Adewole, Olusegun Adeyemo, Clement Adebamowo

**Affiliations:** 1 Department of Geographical Sciences, Center for Geospatial Information Science, University of Maryland, College Park, MD USA 20742; 2 Department of Obstetrics and Gynaecology, University of Medicine Teaching Hospital, Ondo, Nigeria; 3 Center for Bioethics and Research, Ibadan, Nigeria; 4 Department of Epidemiology and Public Health and Greenebaum Comprehensive Cancer Center, Institute of Human Virology, University of Maryland School of Medicine, Baltimore, MD 21201 USA

**Keywords:** Spatial accessibility, Cancer screening services, Health care planning, Nigeria, Low-and middle-income countries

## Abstract

**Background:**

Women in low- and middle-income countries (LMIC) remain at high risk of developing cervical cancer and have limited access to screening programs. The limits include geographical barriers related to road network characteristics and travel behaviors but these have neither been well studied in LMIC nor have methods to overcome them been incorporated into cervical cancer screening delivery programs.

**Methods:**

To identify and evaluate spatial barriers to cervical cancer prevention services in Ondo State, Nigeria, we applied a Multi-Mode Enhanced Two-Step Floating Catchment Area model to create a spatial access index for cervical cancer screening services in Ondo City and the surrounding region. The model used inputs that included the distance between service locations and population centers, local population density, quantity of healthcare infrastructures, modes of transportation, and the travel time budgets of clients. Two different travel modes, taxi and mini bus, represented common modes of transit. Geocoded client residential locations were compared to spatial access results to identify patterns of spatial access and estimate where gaps in access existed.

**Results:**

Ondo City was estimated to have the highest access in the region, while the largest city, Akure, was estimated to be in only the middle tier of access. While 73.5% of clients of the hospital in Ondo City resided in the two highest access zones, 21.5% of clients were from locations estimated to be in the lowest access catchment, and a further 2.25% resided outside these limits. Some areas that were relatively close to cervical cancer screening centers had lower access values due to poor road network coverage and fewer options for public transportation.

**Conclusions:**

Variations in spatial access were revealed based on client residential patterns, travel time differences, distance decay assumptions, and travel mode choices. Assessing access to cervical cancer screening better identifies potentially underserved locations in rural Nigeria that can inform plans for cervical cancer screening including new or improved infrastructure, effective resource allocation, introduction of service options for areas with lower access, and design of public transportation networks.

## Background

Cervical cancer is one of the most common cancers among women worldwide, especially in low- and middle-income (LMIC) countries. The age-standardized incidence of cervical cancer in the World Health Organization (WHO) Africa Region was 33.1 per 100,000 women and the mortality rate was 24.6 per 100,000 women in 2018 compared to the age-standardized incidence of 10.5 per 100,000 women and the mortality rate of 3.8 per 100,000 women in the WHO European Region during the same year [1]. In Nigeria for this same year, cervical cancer was the second commonest cancer among women with an age-standardized incidence of 27.2 per 100,000 women, and mortality rate of 20.0 per 100,000 women [1]. Addressing the burden of cervical cancer is therefore a major public health challenge for Nigeria and other LMICs [2, 3]. With Nigeria expected to become one of the most populous countries in the world by the year 2100 next to only India and China, cervical cancer screening is critical to guard against the expected increase in the burden of this disease [4–6].

Well-organized screening programs can reduce cervical cancer incidence and mortality [7, 8]. Several technologies including cytology (the Papanicolaou smear or Pap smear) and Human Papillomavirus (HPV) DNA test are being used in upper-middle or high income (UMHI) countries [9]. However, in Nigeria, as in other LMIC, there are no successful, widespread, systematic cervical cancer programs [10, 11]. The coverage of cervical cancer screening (i.e., the percentage of female population being screened) is estimated at 8.7% for all women aged over 18 [1, 5, 12–15]. The reasons for this include socio-demographic characteristics (e.g., household income and religion), behavioral factors (e.g., lifestyles and health habits), poor awareness of preventive health services, and barriers to accessing screening services, including spatial factors [16].

Spatial accessibility to healthcare refers to the relative ease with which healthcare services can be reached from a given location and it is increasingly used in public health and epidemiological studies [17]. Spatial access is focused on spatial connectivity and travel impedance between supply (i.e., cervical screening facilities) and demand (i.e., individual residents) [18–21]. One simple way to measure spatial accessibility is to determine the ratio between the supply and demand within a standard area unit. However, this approach does not take variations in distances within the areal unit or the interactions between supply and demand across areal units into consideration. To overcome these limitations, gravity-based methods that consider interactions between supply and demand across different catchment areas have been applied to studies of healthcare access [19, 22]. One special case of the gravity model that has become popular is the Two-Step Floating Catchment Area (2SFCA) model developed by Luo and Wang (2003), which has been extended to a family of Enhanced Two-Step Floating Catchment area (E2SFCA) models by integrating a certain distance decay function, such as a kernel density function or a Gaussian function into the model to account for distance decay effects within each catchment area (i.e., a continuous decrease in the intensity of interaction with increasing distance) [20, 21, 23, 24]. The two key spatial variables in the E2SFCA model are the travel time that determines the catchment area size, and the distance decay function that captures clients’ care-seeking travel behaviors, both of which are assumed to reduce with distance. Building upon the E2FSCA model, more complicated variants have been developed to accommodate different real-world scenarios. For example, the Variable-Width Floating Catchment Area (VFCA) method that assigns flexible catchment sizes to different facilities based on their individual provider types or neighborhood types [25, 26]; the Multi-Mode 2SFCA method that incorporates multiple transportation modes instead of assuming people are traveling to health facilities by a single (or uniform) transportation mode [27]; as well as the 3FSCA method that considers users’ preferences and competition among hospitals [28].

We have several longstanding collaborations with cervical cancers screening sites where we have been examining barriers to cervical cancer screening in Nigeria [29]. In this study, we investigated spatial access to cervical cancer screening services in Ondo State, a predominantly rural state in Nigeria whose socio-demographic and geographic characteristics, makes this region representative of many LMIC cancer screening environments. We studied spatial barriers for clients of the Mother and Child Hospital (MCH) in Ondo City, the only hospital designated as a hospital for women and children in the state, and the only facility offering same day VIA screen and treat in the state at the time the study was conducted. In addition, we analyzed how access varied across the broader region surrounding Ondo City, to inform recommendations for improving the uptake of these services for this part of Ondo State.

For this research, travel mode was an important parameter in the model used to compute spatial access. Nigeria’s car ownership is one of the lowest among LMIC countries [30]. The Nigerian National Bureau of Statistics reported that as of the third quarter of 2017, Nigeria had an estimated 11.5 million motor vehicles in the country, with only an estimated 2.4% of Nigerians owning one private vehicle. Based on this information and with further input from local research team members that hospital clients were more likely to take either shared minibus or taxis to the clinics especially from outlying towns and cities, a multi-modal approach was used for travel modeling. We extended the traditional E2FSCA by integrating multi-modal travel into the model to design a quantitative index for spatial access that was customized for both MCH Ondo City, and the surrounding area in Ondo State. We also took urban and rural differences in travel time estimates into consideration to generate variable catchment sizes for the study region. We investigated spatial access for the specific case of clients accessing cervical cancer screening services at the MCH in Ondo City, as well as for the local population residing in surrounding towns and cities in this part of Ondo State.

## Methods

### Study area

The study area for this research encompasses four cities, Akure (population approximately 424,900 estimated using 2015 LandScan^1^ data analysis), Ondo (population 315,473), Okitipupa (population 108,140), Ore (population 90,401) in Ondo State, a region covering approximately 9500 km^2^ [31]. These cities serve as the main source of clients that have sought cervical cancer screening services at the MCH, Ondo. MCH offered cervical cancer screening using visual inspection with acetic acid and Lugol’s iodine (VIA/VILI) and thermal coagulation for single visit, screen and treat. There were four other hospitals in this area that also offered cervical screening services including State Specialist Hospital Okitipupa (SSH Okitipupa), General Hospital Ore, State Specialist Hospital Akure (SSH Akure), and the State Specialist Hospital Ondo (SSH Ondo). The major form of cervical screening service offered in these hospitals was opportunistic Papanicolaou (Pap) smears for cytology.

### Data

#### Client residential data

**Fig. 1:**
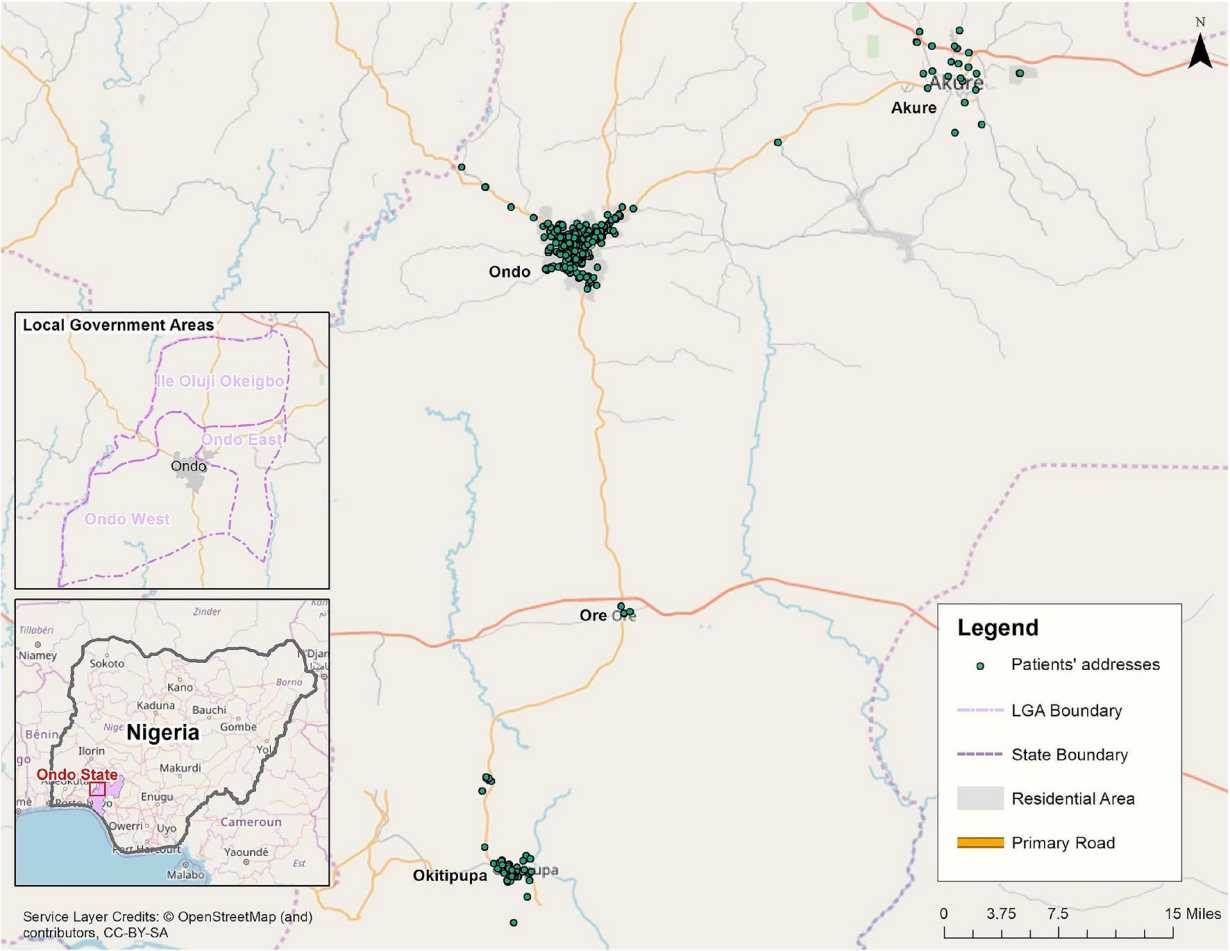
Spatial distribution of residential locations of clients seeking cervical screening service at the MCH in Ondo City

In 2017, we used residential addresses provided by 621 number of clients who received cervical screening at the MCH in Ondo City from 2014 to 2016 for analysis of spatial access to cervical cancer screening in this hospital. In Nigeria, addressing and location reference for postal addresses, house numbering and street naming is poor. In addition, clients may use surrogate locations, such as a church or local business located a short distance of their homes, for their location information. For this reason, specifying and communicating the locations of clients’ residences without a standard street address is challenging. For this research, we used What3Words (W3W) [32], a geocoding system based on random combinations of three words. W3W works especially well for locations where standardized addressing systems do not exist and has a granularity appropriate for addressing tasks in this study (3mx3m grid cells). We used a team of local experts who were familiar with the addresses reported by clients to capture the best estimated location of a client’s reported residence address using W3W. A small number of clients (12 clients) resided outside of the four cities and towns that served as the focus of analysis in this study and were not included in this analysis. Of the remaining addresses, 400 (64%) were successfully converted to geographic coordinates: 73% in Ondo City, 14% in Okitipupa, 8% in Akure, and 2% in Ore (Fig. 1).

#### Cervical screening services

We obtained the number of cervical screening professionals at the MCH and the other hospitals in Ondo State as summarized in Table 1. We obtained and validated the geolocation of the hospitals using Google Maps [33].

**Table 1:** Cervical cancer screening professionals at the MCH and other regional hospitals

Hospital	Number of cervical cancer screening professionals
Mother and Child Hospital Ondo	26
State Specialist Hospital Ondo	7
State Specialist Hospital Akure	10
Specialist Hospital Okitipupa	10
General Hospital Ore	2

#### Population data

**Fig. 2:**
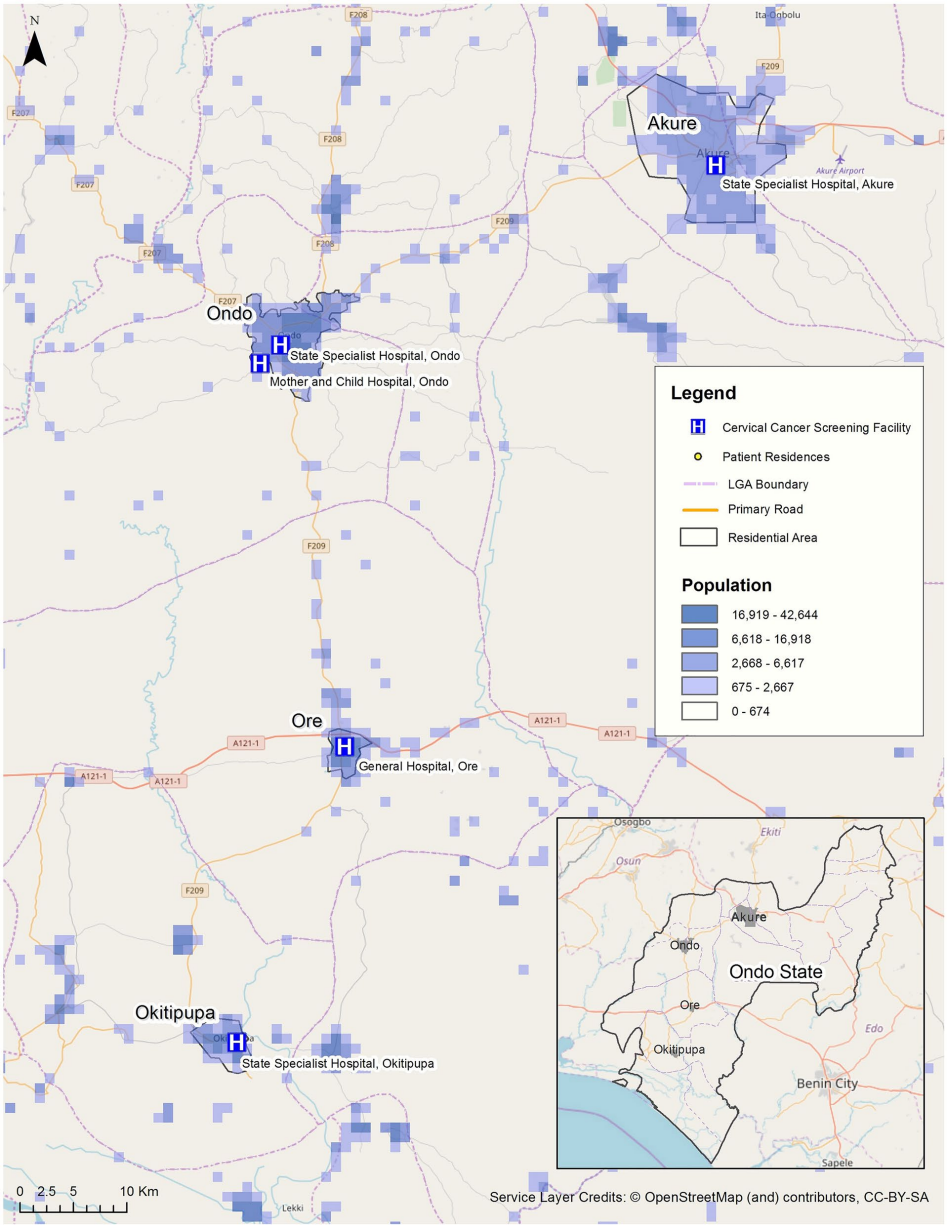
Distribution of local populations in Ondo State, Nigeria

Due to a lack of fine-resolution census-based population data for Ondo State, we used gridded raster population data available through LandScan, a product developed and made available by Oak Ridge National Laboratory (ORNL) [31]. LandScan provides a global population distribution, with approximately 1 km (30″ × 30″) spatial resolution. The population data used in this study was extracted from the 2015 LandScan dataset so as to be a close temporal match to the residential address data used in this study. Population grid cells where residential populations were less likely to have residences (e.g., forested areas identified from Google Maps) were excluded from analysis. Figure 2 highlights the locations of Ondo State population centers for populations larger than 674 in the grid cell (Fig. 2).

#### Road network data

**Fig. 3:**
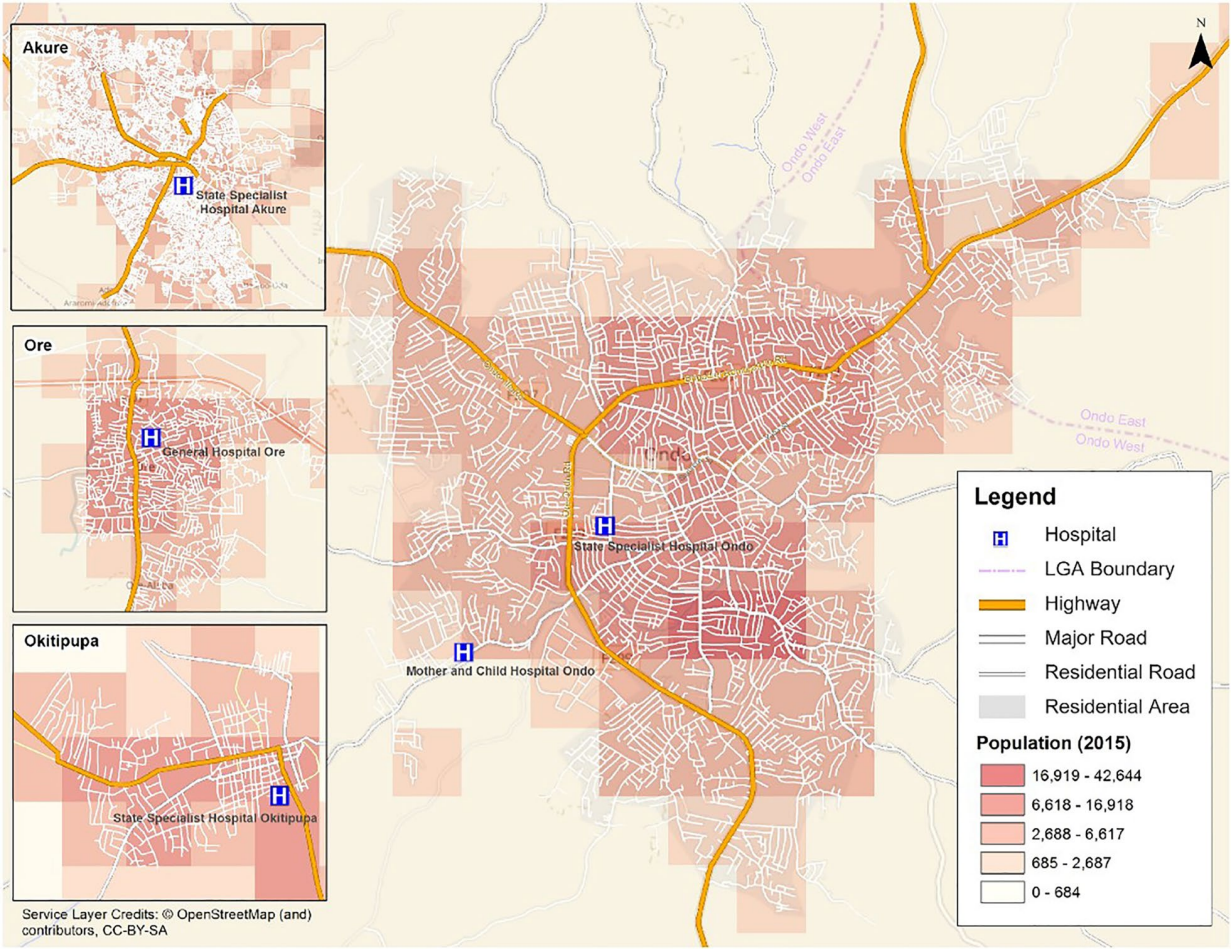
Population, road network coverage, and locations of hospitals with cervical cancer screening clinics in Ondo City, Akure, Okitipupa and Ore

Road network data accessed from OpenStreetMap (OSM) [34] in 2017 was used for computing estimated travel times between the population centers and the cervical cancer screening facilities. The OSM data for this region consisted mainly of highways with a few major roadways, and on its own was inadequate for accessibility analysis at the spatial granularity of town level. We created a road network geodatabase to extend the existing base data by digitizing a more complete road network from 2017 high-resolution satellite image through Bing Maps service [35]. A total of 5615 road segments were digitized and a topological check was performed to correct any topology errors that existed in the original OSM data or were created during the digitization process. The result produced 2970 road segments in Akure, 1621 in Ondo City, 632 in Ore, and 392 in Okitipupa (Fig. 3). These roads were then reclassified as highways, major roads and residential roads based on visual inspection from the satellite imagery as well as existing road classifications by Google Hybrid Maps. Highways represented higher traffic volume roads that connected the major cities. Major roads fed into highways or were paved, wide streets detected in the cities. Local roads that connected residential areas were classed as residential roads.

**Fig. 4:**
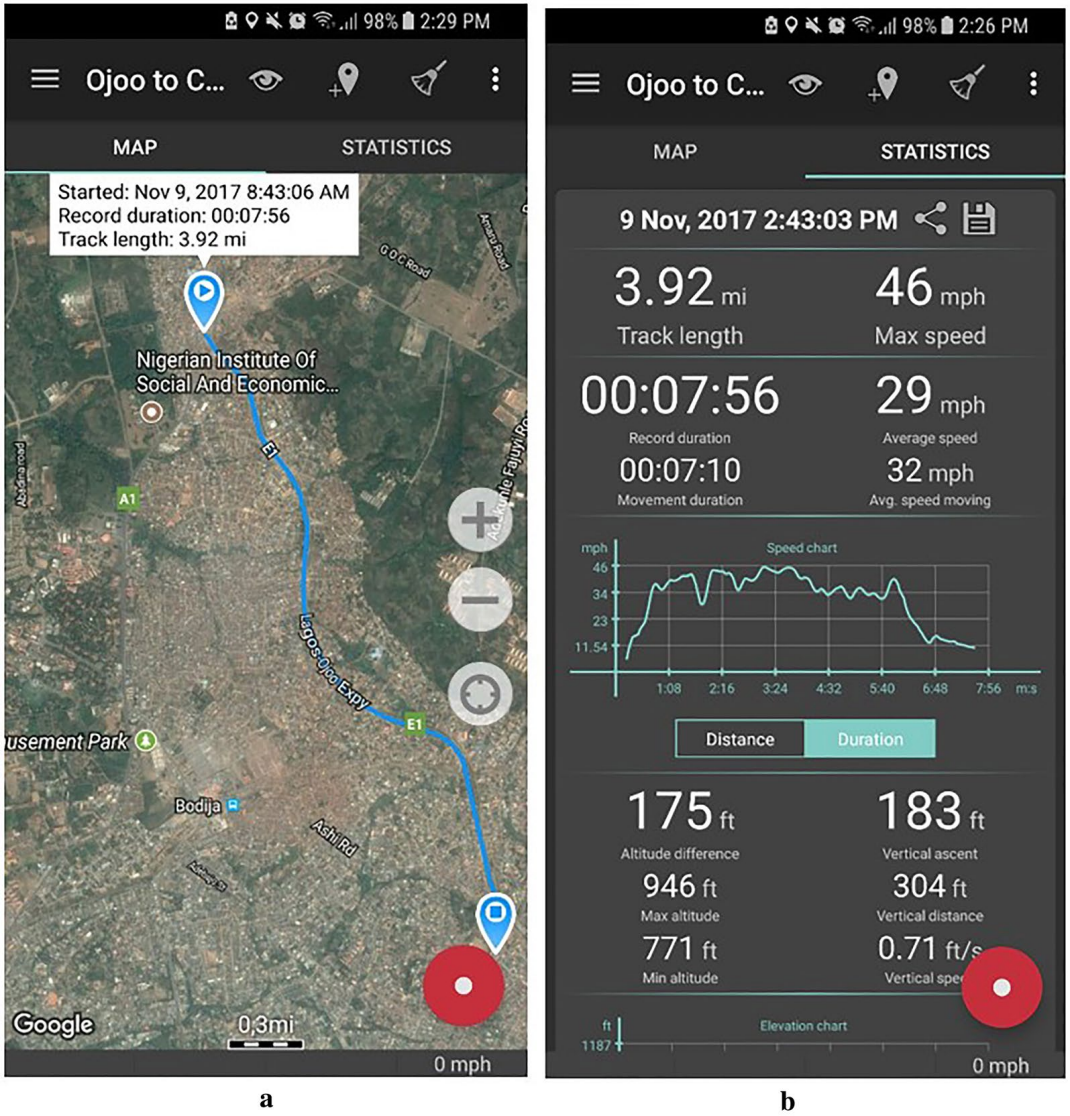
User interface for the Geo Tracker app that was used to record road network attributes for representative roads showing **a** the map interface and **b** speed and distance diagnostics

Each road was assigned a value for travel time based on the length of the road segment and an estimated travel speed by road type. The estimates of travel speed for representative roads of each type in each city were collected locally using GPS tracking data collected using the Geo Tracker [36] smart phone application, a GPS track recording app for Android phones. This app allowed us to collect travel speed information as tracks saved to phones locally and displayed on the in-app map as shown in Fig. 4a for one such track recorded by the local team when they were driving on the Lagos-Ojoo Expressway (Fig. 4a). Additional track information including track length, maximum and average speed, tracking duration, as well as a speed chart could be found in the statistics tab of the app (Fig. 4b).

The travel speeds were collected during estimated time windows when hospital clients would be typically travelling to care (e.g., to arrive at a clinic by 8am local time, prior to the typical 9am time when clinics open for the day). Scheduled appointments were not the norm in this area, and clients tended to arrive early in order to be sure of securing an appointment. The travel speeds for each road type were tracked (Table 2), and the mean speed of travel for the collected tracks for each type of road was used in the model. The travel speed for the four cities depended on traffic volume and local road conditions. While there were some variations noted in our testing, overall, the mean travel speeds for highways were higher than for major roads and residential roads.

**Table 2:** Estimated mean travel speeds (km/hr) for three types of roads in Ondo City, Akure, Okitipupa, and Ore

Cities	Highways	Major roads	Residential roads
Ondo City	53.00	38.00	29.80
Akure	37.00	48.67	27.40
Okitipupa	53.33	31.00	11.35
Ore	63.00	17.68	7.78

### Analysis

#### Modeling spatial access to cervical cancer screening services

The spatial access index of the original Two-step Floating Catchment Area (2SFCA) model is represented by the physician-to-population ratio that is calculated by repeating the process of creating “floating catchment areas” twice (once on supply locations and once on demand locations) that are also within certain threshold travel times [20]. The index created by the 2SFCA is easy to interpret. By utilizing drive time analysis, 2SFCA embeds driving obstacles and distance impedances, and as such is an improvement over using simple buffer zones. The method considers the interaction between supply and demand and saw much use especially in early studies that evaluated healthcare accessibility to physicians. Over time the fact that it assumed equal accessibility among all locations within each catchment (e.g., there is no difference in terms of accessibility between a 10-min drive and a 25-min drive in a 30-min catchment) [23, 37] opened the door for extensions and other approaches. The Enhanced Two-Step Floating Catchment Area (E2SFCA) model assigns weights to different travel time zones to account for distance decay effects within each catchment area [21]. If the supply location is further away from the demand location, it is assumed that there is a lower chance or higher impedance that this specific healthcare provider will be selected for use. Similar to 2SFCA, E2SFCA consists of two steps and in each step, the area within the critical distance is gradually penalized by distance. The distance decay effect is an inverse power function or a negative exponential function. There are a variety of decay functions based on different model assumptions, where it can be a continuous function, a discrete variable, or a hybrid of the two [18, 37, 38].

By generalizing the distance decay as a term $f\left( d \right)$, the E2SFCA is expressed in a model as:

1 $$A_{i} = \sum\limits_{j = 1}^{n} {\left[ {S_{j} f\left( {d_{ij} } \right)/\left( {\sum\limits_{k = 1}^{m} {P_{k} f\left( {d_{kj} } \right)} } \right)} \right]}$$

where *S*_*j*_ is the capacity (number of physicians) for health care provider *j*, *P*_*k*_ is the population at location *k*, $n$ and $m$ are the total numbers of provider locations and population centers, respectively [37].

The E2SFCA model typically assumes only one type of transportation, i.e., passenger vehicles. This assumption is not always true, of course, particularly in LMIC countries where car ownership is relatively low and people use a variety of transportation modes to seek healthcare. An expansion, the Multi-Mode 2SFCA, includes other modes of transportation [27] where this model incorporates $n$ ($n \ge 1$) transportation modes $\left\{ {M_{1} ,M_{2} , \ldots ,M_{n} } \right\}$ and each population $P_{k}$ at location $k$ is divided into multiple subpopulations (where every subpopulation has a single transportation mode), denoted as $P_{k} = \left\{ {P_{{k,M_{1} }} ,P_{{k,M_{2} }} , \ldots ,P_{{k,M_{n} }} } \right\}$. The first step is adding the subpopulation structure to the traditional 2SFCA and physician-to-population ratios ($R_{j}$) are formulated as:

2 $$R_{j} { = }\frac{{S_{j} }}{{\sum\limits_{{k \in \left\{ {d_{kj} (M_{1} ) \le d_{0} (M_{1} )} \right\}}} {P_{{k,M_{1} }} } + \sum\limits_{{k \in \left\{ {d_{kj} (M_{2} ) \le d_{0} (M_{2} )} \right\}}} {P_{{k,M_{2} }} } + \cdots + \sum\limits_{{k \in \left\{ {d_{kj} (M_{n} ) \le d_{0} (M_{n} )} \right\}}} {P_{{k,M_{n} }} } }}$$

where $d_{kj} (M_{n} )$ is the travel time for travel mode $M_{n}$ between population location $k$ and provider location $j$. $d_{0} (M_{n} )$ is a predefined threshold travel time from $j$ by mode $M_{n}$. As a second step, the model is further augmented by considering multiple transportation modes and Multi-Mode 2SFCA is then expressed as:

3 $$A_{i} = \frac{{P_{{i,M_{1} }} \sum\limits_{{j \in \left\{ {d_{ij} (M_{1} ) \le d_{0} (M_{1} )} \right\}}} {R_{j} + P_{{i,M_{2} }} \sum\limits_{{j \in \left\{ {d_{ij} (M_{2} ) \le d_{0} (M_{2} )} \right\}}} {R_{j} } + \cdots + P_{{i,M_{n} }} \sum\limits_{{j \in \left\{ {d_{ij} (M_{n} ) \le d_{0} (M_{n} )} \right\}}} {R_{j} } } }}{{\sum\limits_{r = 1}^{n} {P_{{i,M_{r} }} } }}$$

where the physician-to-population ratios ($R_{j}$) of each provider location are weighted by the size of the subpopulations by catchment areas then summed to calculate an accessibility index ($A_{i}$) [27]. In this way, the accessibility of a location is dependent not only on the distribution of the provider facilities around it, but also on its subpopulation structure and their corresponding transportation modes.

To design the model used in this research, we combined components from the Multi-Mode 2SFCA and the E2SFCA and used this hybrid model (referred here as Multi-Mode E2SFCA) to model spatial access for both the MCH Hospital cervical cancer screening clients and more generally, for the population in the towns and cities surrounding Ondo City. For this analysis, we included different travel assumptions for urban and rural locations identified based on population and land cover information. In the study area, there were cities with higher populations (the state capital Akure, Ondo City, Okitipupa, and Ore) surrounded by expansive rural areas that were mainly forested and where the residential settlements were scarcer and more dispersed. Thus, although assigning a uniform catchment size (e.g., 30 min travel time) could be suitable for case studies in UMHI countries where urbanization is more present, and where there are well-constructed road infrastructures, it does not capture the sparser populations in rural regions in LMIC, and could result in an underestimation of access for travel over longer distances by completely cutting them off [20, 21, 38]. To solve this problem, we used a classification approach applied in a previous study on urbanization and urban expansion in Nigeria, where settlements were classified as urban if they comprised 20,000 people or more [39], and classified the study area into urban and rural land use types using 2015 LandScan population estimates. All four cities in our study area were classified as urban while the regions outside the city boundaries were classified as rural. Different catchment sizes were assigned to urban and rural locations and adjusted based on input and insights from the local team in Nigeria about the maximum and average travel times of clients at the regional hospitals. In general, catchment size tended to be larger in rural areas, and smaller in urban areas reflecting the case that rural residents were likely to travel longer distances to seek care in the cities [21, 40].

To differentiate accessibility within a catchment, we used a Gaussian distance decay function [41–43]. The distance decay term $f\left( d \right)$ was formulated as:

4 $$f\left( {d_{ij} } \right) = e^{{ - d_{ij}^{2} /\beta }}$$

where $d_{ij}$ was the travel time between population center location $i$ and provider location $j$. The travel times were computed using the road network dataset and an Origin–Destination Cost Matrix generated using the Network Analyst Extension of ArcGIS 10.5 based on the computed road network distances and collected travel speeds. The value of the impedance coefficient $\beta$ in this study was determined by calculating the value at which the Gaussian curve approached zero [36, 38]. Two different values $\beta_{urban}$ and $\beta_{rural}$ were calculated for urban and rural locations based on their respective catchment sizes and using threshold travel times of $d_{0,urban} = 40\hbox{min} s$ and $d_{0,rural} = 1.5hrs$ respectively. The model was implemented in R, an open source programming software environment for data analysis.

The model was extended to incorporate two travel modes used by clients accessing cervical cancer screening at the clinics in this region. We incorporated the two most popular modes of travel—taxi and mini bus—in the model. To integrate these two transportation modes and to better represent the different travel mode choices of urban and rural residents, we assigned the proportions of two transportation modes to each subpopulation group in the Multi-Mode E2SFCA. A Gaussian distance decay function was included in the model as described above to account for catchments change with distance. Based on local information, we used the assumption that urban residents were more likely to take taxis (80%) to the hospitals and the minority (residents living in the outskirts of the city) would take a minibus (20%), while the majority of rural residents were more likely to take minibus (80%), and fewer people in rural areas would take taxis (20%) for longer distance travel to the hospital screening centers.

We undertook a sensitivity test to compare the spatial access indices calculated by our model using different proportions of travel modes to test the sensitivity of these choices on the spatial access results. We tested the model with different assumptions including 80%/20% (taxi/minibus), 70/30, and 60/40 for urban residents, and similarly tested the opposite proportions of travel modes (e.g., 80%/20% minibus/taxi) for rural residents. Considering the fact that shared minibus is picking up and dropping off multiple passengers at several locations during the trip, we added a 15-min time penalty to the total travel time for trips estimated to be made by minibus.

## Results

**Fig. 5:**
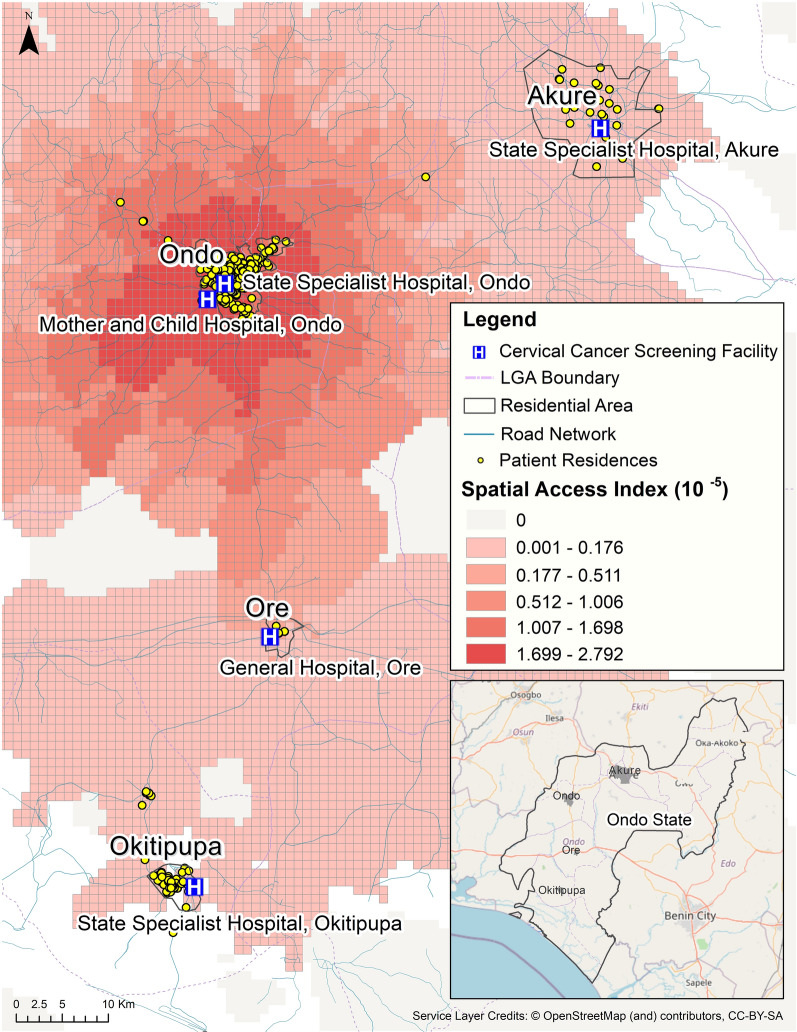
Comparing MCH client residential locations with modeled spatial access for cervical cancer screening services at MCH Ondo City

We investigated spatial access for clients of MCH in Ondo City and the surrounding cities and towns in Ondo State. To investigate spatial access for clients of MCH in Ondo City, the model used MCH as the designated cervical cancer screening facility. The geocoded residential locations of MCH clients were combined with the modeled access surface to provide more insights about the distribution of demand and the impact of geographic factors on access to the cancer screening clinic at this hospital (Fig. 5).

The results of this analysis showed that while 73.5% of MCH Ondo clients resided in the two highest access zones, 21.5% of clients were from locations estimated to be in the lowest access catchment, and a further 2.25% of the clients were living beyond any of the access catchments, i.e., had very poor spatial access. The analysis found that Akure (the largest city in this region) was home to 7.5% of MCH clients with geocoded residences and was located within the second lowest zone of access. There was also sizable group of residents (14.25%) in Okitipupa (the second largest population center in the area) who were clients of MCH and who traveled the farthest distances. These individuals resided beyond the catchment area of MCH with poor spatial access. The city of Ore, home to 1.75% of clients with geocoded residences, had better access than the Okitipupa clients due to better road coverage and a shorter distance to MCH.

We analyzed the estimated travel time from the client addresses (69% MCH clients resided in urban areas and 31% resided in rural areas) to MCH and found that 75% of clients could access MCH within 40 min by taking taxis, and 77% of clients could access MCH within 1.5 h by taking a minibus.

**Fig. 6:**
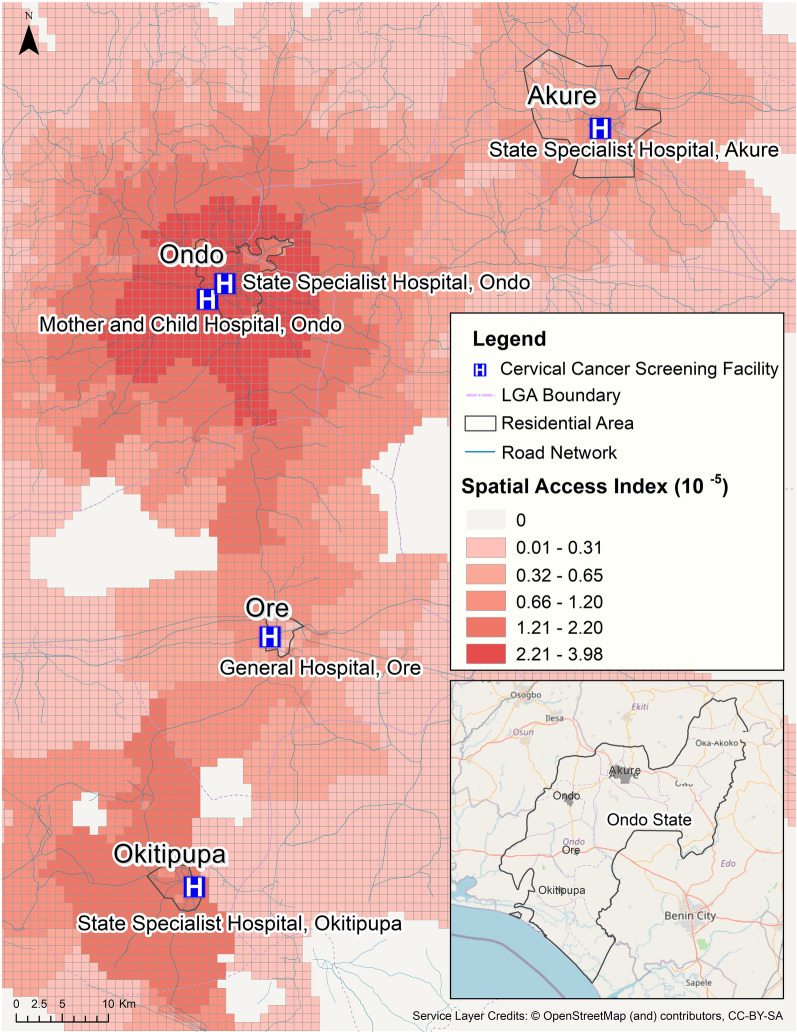
Spatial access surface for cervical cancer screening services for the area surrounding Ondo City in Ondo State, Nigeria

We implemented the Multi-Mode E2SFCA model to compute the spatial access index for the healthcare infrastructures in the cities and towns surrounding Ondo City. For this modeling task, we calculated the distance decay parameters for travel, and represented each of the population centers by the centroids of the LandScan population grid cells. The estimated travel time from each population center to the closet cervical cancer screening service was calculated based on the length of the road segments and estimated travel speeds by road type. We found that while urban residents were estimated to be able to access cervical cancer screening services within 40 min by taking either taxi or mini bus, only 52% of rural residents were estimated to be able to access the services within 40 min by taking taxis. Model results showed that 97% rural residents had access within 1.5 h by taking a minibus. The resulting spatial accessibility surface for these population centers was classified using natural breaks into 6 access zones, capturing access to cervical cancer screening services for the local populations in the cities and towns surrounding Ondo City (Fig. 6). Ondo City and its suburbs were located in the highest access class in the region, with two cervical cancer screening facilities (MCH and SSH Ondo), and the highest number of cervical cancer screening professionals. Although the access index mostly decreased gradually along the road network from each of the hospitals with cervical cancer screening facilities outwards, there existed locations that were relatively close to the services but with lower access values due to sparser road network coverage and fewer options for public transportation. Notably, the area near Okitipupa fell into the second highest access class (approximately a 1.5-h drive to Ondo City) while Akure, which had a similar number of cervical cancer screening professionals and was closer (approximately 40-min drive) to Ondo City’s screening services, was only in the 3^rd^ highest access class. This can be explained by the large difference between these two cities’ demand for the service—as Ondo State’s capital city, Akure has an estimated population of 424,900, which is almost four times of the population size of Okitipupa (108,140). The city of Ore was estimated to be in the lowest access class due to the small number of cervical cancer screening professionals in the General Hospital, Ore (low contribution on the supply side) even given its smaller population size among these four cities.

Sensitivity tests were implemented to understand how applying different assumptions about the travel modes in the Multi-Mode E2SFCA might affect the spatial access results. The sensitivity test found that for urban residents, the model uncertainty possibly caused by selecting a particular proportion of travel modes was minimal. 32.7% of urban residents were estimated to have high access (i.e., in the first and second highest access classes of zones) when selecting 80%/20% taxi/minibus. This percentage held constant when using 70%/30% taxi/minibus, and slightly decreased to 31.8% when selecting 60–40 split for taxi/minibus. This was likely due to the relatively shorter travel distances for residents in cities or towns and their suburbs. For rural residents, however, the sensitivity analysis demonstrated that spatial accessibility increased as the percentage of taxi trips was increased. For example, when the proportion increased from 20% taking taxis to 40%, rural residents that were estimated to be in the lowest and second lowest access zones decreased by 32.6% and 4.8%, respectively. Testing with higher percentages of rural residents that were likely to take taxis in the model resulted in less populations with low access. However, given that travel distances to cervical cancer screening services for rural residents were longer, and taking taxis was more costly, we know that this last test doesn’t capture actual local travel behavior, and the model assumptions of 20/80 (taxi/minibus) for rural residents more closely represents actual travel patterns.

## Discussion

In this research project, we developed a Multi-Mode E2SFCA index for measuring and visualizing spatial access to cervical cancer services in Ondo State, Nigeria. We identified locations, particularly for clients residing in the town of Okitipupa, where spatial access could present a challenge and impact those seeking care or following through on subsequent appointments. These findings fit with the results from a study identifying different kinds of barriers to providing quality cancer care in Nigeria, where key deficiencies reported were lack of physicians and trained staff, and the necessary diagnostic treatment equipment at hospitals causing clients to travel further to other locations for care [44]. More client-centric approaches to delivery of cancer care was also noted in [45] where client-family hostels providing temporary accommodation, and reduced transport fees were reported as efforts being considered to reduce barriers of access to cancer care.

The data collection and modeling framework proposed in this paper should also generalize well for studies of spatial access to healthcare services in other LMICs. How spatial access varies across a region can provide useful insights for improved and more efficient healthcare planning and resource allocation for cancer screening services in an African LMIC, where, in general, few studies on cancer patient navigation interventions have been undertaken [46]. The approach we used in this study can also be generalized to other health disciplines in other LMICs where framework data such as road network data and client addressing are not readily available or easy to geocode [35, 47, 48].

This analysis focused on one hospital where client address information was available. While information from a single hospital could be viewed as a potential limitation, it is important to point out that the MCH in Ondo City was the only provider of VIA based, same day, cervical cancer screen and treat program in the region at the time of this study. Our results can assist decisionmakers with determining areas with the least access to existing services to inform the location of new services. In addition, our estimates for types of road (the road classification) and travel speeds have some uncertainty associated with them as we could only test a subset of the road network with local drivers, and collect data using the GeoTracker app. We applied our reasoning to all the cities and towns in the study area, which may be an oversimplication and could result in both over and underestimation of travel speeds due to local conditions. As road network data become more available in LMICs due to the more widespread adoption of navigation apps, this limitation should be reduced in future research.

Future work could expand on the number of hospitals, and also address topics such as extending the types of potential travel modes beyond the two transportation modes, taxi and minibus, that we focused on in this study to include other modes (e.g., other buses and walking). The information provided to us by local experts suggested these two travel modes were commonly used in the Ondo City area but collecting clients’ travel behavior explicitly through the use of GPS devices, apps, or travel survey, or being able to access public transit data could add useful insights into travel behaviors [49, 50]. We know that in reality, some urban residents may choose to walk to close-by hospitals due to proximity so including pedestrian travel could be relevant. Another topic that is key for spatial access estimations relates to estimates for travel speed on different road types. As data on driving speeds was not readily available (posted speed limits were not common in this area), we assumed that all the roads of the same type or category had the same travel speed at all times. However, road conditions, times of travel and traffic all impact the actual travel speed on a road network. More information on travel speed would be helpful to highlight areas where traffic impacts access more heavily. Future research could also investigate extending the model based on more realistic estimates of the utilization of screening services accounting for variations in quality, variety and the cost of services that contribute to the relative attractiveness of different service facilities in Nigeria or other LMICs.

## Conclusions

Access to healthcare services will continue to be a key topic in research on public health, especially in the context of the increasing social health inequalities in Nigeria and other low- and middle-income countries. We used a hybrid approach referred to as the Multi-Mode Enhanced Two-Step Floating Catchment Area (Multi-Mode E2SFCA) model in this study to determine spatial access to cancer screening services in the region of Ondo City, Nigeria. This model incorporated a number of different factors to provide a more realistic and comprehensive measure of access including variable catchments computed using distance decay effects, an accounting for both urban and rural areas, and multiple modes of travel (taxi and mini bus), that helped to better identify locations that may be underserved. Sensitivity analysis for the model showed that spatial access results for rural locations were sensitive to the choice of travel mode. This is especially relevant for healthcare providers to effectively plan for more accessible and equitable access to cancer screening services, and to strategically invest in transportation infrastructures in vulnerable areas with low spatial accessibility. Reducing spatial barriers could also help to reduce the severity of disease or the stage at which the disease is diagnosed. The methods used in this study for geocoding, travel speed modeling, and estimating population size–key elements for spatial access modeling–are generalizable to other low- and middle-income countries that share similar characteristics of addressing, transportation conditions, population distributions, and expanding urbanization.

## Data Availability

The road network dataset used during the current study can be available upon reasonable request. The population data used in this project are available from the LandScan™ data repository (http://web.ornl.gov/sci/landscan/index.shtml) free of charge for U.S. Federal Government Agencies, all users must register to access the data set. The client address dataset generated during the current study is not publicly available due to privacy issue.

## Abbreviations

2SFCA: Two-Step Floating Catchment Area
E2SFCA: Enhanced Two-Step Floating Catchment Area
LMICs: Low- and middle-income countries
MCH: Mother and Child Hospital Ondo
SSH: State Specialist Hospital

## Footnotes

1 LandScan: https://landscan.ornl.gov/.
